# A novel mutation in *unc-112*/kindlin locus causes distal tip cell migration defects

**DOI:** 10.17912/micropub.biology.000265

**Published:** 2020-06-08

**Authors:** Aileen Park, Zhongqiang Qiu, Josh Bumm, Myeongwoo Lee

**Affiliations:** 1 Department of Biology, Baylor University, Waco, Texas 76798, United States; 2 Baylor University Department of Biology

**Figure 1:**
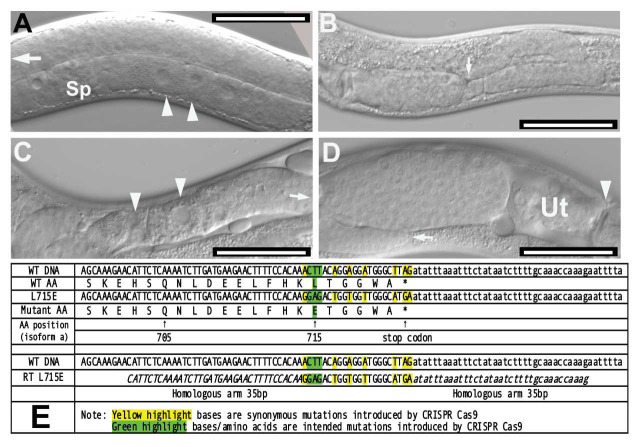
**A**. N2 *Caenorhabditis elegans* displaying normal gonad. White arrowheads indicate developing oocytes in the ventral side of the organism (proximal gonad). The distal gonad is filled with undifferentiated germ cell nuclei, white arrow. Sp: spermatheca. Bar = 50 μm. **B**. *unc112(kq715)* mutant displaying distal tip cell migration defects (DTC Mig). White arrow indicates abnormal “dipping” of the distal arm. Bar = 50 μm. **C**. *unc-112(kq715)* mutant displaying ectopic oocytes. White arrowheads indicate meiotic oocytes developing in the distal gonad arm. Arrow indicates the direction of DTC. Bar = 50 μm. **D**. *unc-112(kq715)* mutant displaying abnormal bulging gonad filled with undifferentiated germ cells between label D and uterus (Ut). Bar = 50 μm. **E**. Comparison of wild-type *unc-112* amino acid sequence and the mutant sequence including the repair template sequence and synonymous mutation schemes. All sequences are written in the 5’ to 3’ direction. *WT: wild-type. **AA: amino acid.

## Description

The *unc-112* gene in *Caenorhabditis elegans* encodes a 720-amino acid protein (UNC-112) that colocalizes with integrin and perlecan in cell to ECM adhesion structures and is a component of dense bodies and M-lines (Rogalski **et al.*,* 2000). The UNC-112 protein is homologous to the human protein Kindlin-1. Kindlin-1 in humans is implicated in Kindler syndrome, a skin fragility disorder (Siegel **et al.*,* 2003). The UNC-112 protein shares a short region of homology (roughly 200 amino acids) with talin and other FERM superfamily proteins that may play a key role in plasma membrane attachment (Rogalski **et al.*,* 2000). Previous studies have discovered that UNC-112 is essential for the organization and localization of PAT-3/β-integrin in body wall muscle cells (Rogalski *et al.*, 2000). Null mutations in *unc-112* lead to twofold stage arrest during embryogenesis, abnormal body wall muscle, and exhibit the Pat (paralyzed, arrested elongation at twofold) phenotype (Williams and Waterston, 1994). In this study, a novel mutant allele of *C. elegans unc-112* was generated using CRISPR-Cas9 gene editing. In this mutant allele, leucine (L) was replaced with glutamate (E) near the C-terminal end of UNC-112. In a biochemical study, the homologous mutation abolished binding of kindlin2 to the membrane distal “phospho-tyrosine” motif of β1 integrin tails (Li **et al.*,* 2017). Mutant worms were examined for behavioral and morphological abnormalities. Mutants displayed no notable behavioral abnormalities in a thrashing assay compared to N2 wild-type worms (p=0.5485 according to the Mann-Whitney U Test). However, 35.6% of *unc-112* mutants (n=177) were observed to exhibit defective distal tip cell migration (DTC Mig). Mutants displaying this abnormal DTC Mig phenotype had dipping distal gonad arms (Figure 1B), bulging proximal gonads filled with germ cell nuclei (Figure 1D), and oocyte-like nuclei in the distal gonad (ectopic oocytes, Figure 1C). Previous research has shown that integrin-ECM interactions play important roles in directing DTC migration and gonad morphogenesis, and the DTC Mig phenotype observed could be the result of mutant *unc-112* disrupting key integrin functions in the somatic gonad (Lee **et al.*,* 2001)

## Methods

In order to induce the desired mutation to *unc-112*, the CRISPR guide RNA Selection Tool (http://genome.sfu.ca/crispr/) was used to identify CRISPR target sites within the gene. The mutant line created, *unc-112(kq715)*, contains a mutation from Leucine to Glutamate at amino acid number 715 near the C-terminal end of UNC-112. Custom template DNA (Temp-4UNC112L715E, Figure E), custom crRNA (UNC112STOP), tracrRNA (cat. no. 1073190), and Alt-R Cas9 nuclease (cat. no. 1081058) were annealed at room temperature (Paix *et al.*., 2015) and micro-injected into the syncytial gonad arms of N2 wild-type worms (P0) (Mello *et al.*, 1991). F1 dumpy (Dpy), co-CRISPR phenotype, worms were isolated and PCR screened for the presence of the desired mutation. The offspring from F1 heterozygous mutants were then PCR screened to isolate homozygous mutants (F2). The isolated mutant line was backcrossed (2x) to N2 and were then studied for phenotype and behavioral characterization. In order to identify DTC Mig phenotypes, N2 and *unc-112(kq715)* worms were self-fertilized and screened for aforementioned distal tip cell abnormalities. Briefly, animals were mounted and observed on a Nikon Eclipse Ni-U compound microscope and images were captured using NIS Elements software (version 5.02). The *unc-112* mutant animals with DTC migration defects were examined with Nomarski optics. The morphology of U-shaped gonad arms was observed in gonad arms at the L4 or young adult stage of hermaphrodites. The percentage of abnormal gonad arms, displaying no looping back, zigzag distal arms, or extra turns were calculated (Lee and Cram, 2009). The thrashing assay was performed by counting the number of body bends over a 10 second period when suspended in a 10 μl drop of M9 buffer (Lee *et al.*, 2005). A Mann-Whitney U Test was then run to confirm statistical significance of thrashing assay results

**crRNA sequence**

UNC112STOP: CCACAAACTTACAGGAGGAT

**PCR Primers**

UNC112L715SEQF: AACTTGGTGCTGACAGGAAGG

UNC112L715SEQR: GCGAGATATGCGAAACGTTGA

UNC112L715WTR: ATCTAAGCCCATCCTCCTGTAAGT

UNC112L715ER: TCATGCCCAACCACCAGTCTCC

## Reagents

BU0715 *unc-112(kq715)* is available upon request.

## References

[R1] Lee M, Cram EJ, Shen B, Schwarzbauer JE (2001). Roles for beta(pat-3) integrins in development and function of Caenorhabditis elegans muscles and gonads.. J Biol Chem.

[R2] Lee M, Shen B, Schwarzbauer JE, Ahn J, Kwon J (2005). Connections between integrins and Rac GTPase pathways control gonad formation and function in C. elegans.. Biochim Biophys Acta.

[R3] Lee M, Cram EJ (2009). Quantitative analysis of distal tip cell migration in C. elegans.. Methods Mol Biol.

[R4] Li H, Deng Y, Sun K, Yang H, Liu J, Wang M, Zhang Z, Lin J, Wu C, Wei Z, Yu C (2017). Structural basis of kindlin-mediated integrin recognition and activation.. Proc Natl Acad Sci U S A.

[R5] Mello CC, Kramer JM, Stinchcomb D, Ambros V (1991). Efficient gene transfer in C.elegans: extrachromosomal maintenance and integration of transforming sequences.. EMBO J.

[R6] Paix A, Folkmann A, Rasoloson D, Seydoux G (2015). High Efficiency, Homology-Directed Genome Editing in Caenorhabditis elegans Using CRISPR-Cas9 Ribonucleoprotein Complexes.. Genetics.

[R7] Rogalski TM, Mullen GP, Gilbert MM, Williams BD, Moerman DG (2000). The UNC-112 gene in Caenorhabditis elegans encodes a novel component of cell-matrix adhesion structures required for integrin localization in the muscle cell membrane.. J Cell Biol.

[R8] Siegel DH, Ashton GH, Penagos HG, Lee JV, Feiler HS, Wilhelmsen KC, South AP, Smith FJ, Prescott AR, Wessagowit V, Oyama N, Akiyama M, Al Aboud D, Al Aboud K, Al Githami A, Al Hawsawi K, Al Ismaily A, Al-Suwaid R, Atherton DJ, Caputo R, Fine JD, Frieden IJ, Fuchs E, Haber RM, Harada T, Kitajima Y, Mallory SB, Ogawa H, Sahin S, Shimizu H, Suga Y, Tadini G, Tsuchiya K, Wiebe CB, Wojnarowska F, Zaghloul AB, Hamada T, Mallipeddi R, Eady RA, McLean WH, McGrath JA, Epstein EH (2003). Loss of kindlin-1, a human homolog of the Caenorhabditis elegans actin-extracellular-matrix linker protein UNC-112, causes Kindler syndrome.. Am J Hum Genet.

[R9] Williams BD, Waterston RH (1994). Genes critical for muscle development and function in Caenorhabditis elegans identified through lethal mutations.. J Cell Biol.

